# Enhancements Needed in GE Crop and Food Regulation in the U.S.

**DOI:** 10.3389/fpubh.2016.00059

**Published:** 2016-03-31

**Authors:** Charles Benbrook

**Affiliations:** ^1^Benbrook Consulting Services, Troy, OR, USA

**Keywords:** Coordinated Framework, substantial equivalence, resistance management, gene editing, scientific integrity, resistant weeds, labeling GE food

## Abstract

Genetically engineered (GE) crops, multi-ingredient foods derived from one or more GE ingredients, and GE agricultural inputs are regulated in the United States under a “Coordinated Framework” that was literally cobbled together in the early 1990s. *Via* this Framework, responsibility is spread across three federal agencies for the assessment and management of potential risks arising from the planting of GE crops, the raising of GE animals, or uses of GE inputs. The Framework was incomplete and conceptually flawed from the beginning. Despite multiple, piecemeal efforts to update aspects of GE risk assessment and regulatory policy, the Coordinated Framework survives to this day largely unchanged. Its shortcomings are recognized in both the scientific and legal communities, but meaningful reforms thus far remain out of reach, blocked by the intense controversy now surrounding all things biotech. Five generic reforms and another five specific initiatives are described to create a more robust, science-driven GE regulatory infrastructure in the U.S.

Over most of the last 20 years, the limitations of the Coordinated Framework (1), and the U.S. government’s lax approach to genetically engineered (GE) risk assessment, triggered deep-set concern and scrutiny among some stakeholders and consumers, food companies, and organizations but not much beyond that. In the last 5–10 years though, the slice of U.S. and global markets responsive to concerns regarding the safety, environmental impacts, and/or the socioeconomic consequences of GE crops and inputs has grown, and is now driving economically meaningful shifts in market share (2).

Given that GE applications are now spreading to fresh fruits and vegetables and animals, the range of potential risks and gaps in risk-assessment science are likely to become both more acute and undeniable. At some point, U.S. Ag Inc., and especially those companies and growers significantly dependent on exports, will no longer accept the collateral market damage caused by the shortcomings of the Coordinated Framework and the corollary erosion of confidence in the science supporting the regulation of agricultural biotechnology in the U.S.

Recognizing the growing demand for constructive change, the Obama Administration announced in 2015 that it would undertake a long-overdue review of the Coordinated Framework (3, 4). Their goal is to identify at least some improvements that would garner widespread support and could be implemented *via* Executive Orders and/or regulatory policy changes prior to the transition to a new Administration in January 2017. As part of this ongoing process, the Food and Drug Administration (FDA) issued a notice calling for public comments under the ponderous title: “*Clarifying Current Roles and Responsibilities Described in the Coordinated Framework for the Regulation of Biotechnology and Developing a Long-Term Strategy for the Regulation of the Products of Biotechnology*” (5).

Such an Executive Branch review will hopefully guide the actions of this and the next Administration, as well as Congress, in providing federal agencies a clear mandate and stronger authority to conduct state-of-the-art risk assessments on GE plants and foods, animals, and microbes. Herein, I describe current agency roles and the most important reforms that are needed if this effort is to bear fruit worth harvesting.

## Agency Roles and Responsibilities

### The Food and Drug Administration

The FDA was given responsibility for assessment of food safety risks and most aspects of food labeling, drawing primarily on the Food, Drug, and Cosmetic Act (FDCA). Agency regulations, data requirements, and decision criteria are, in turn, grounded in legislation crafted and passed years before the first applications of genetic engineering in the food industry and agricultural sector. While the FDA’s role within the Coordinated Framework is arguably the most important in terms of protecting public health, its role and actions have for the most part flown below the radar.

The FDA regulates GE animals as new animal drugs, for which there is a mandatory, FDCA requirement for a safety assessment. For GE plants, FDA regulates them under a 1992 Statement of Policy that asserts that GE (a) is just an extension of conventional breeding, (b) does not raise new health risks, and (c) does not need any special safety assessments once nutritional and compositional “substantial equivalence” is demonstrated (6).

There is only a cursory agency review of industry-submitted documents over the course of a “voluntary consultation” (7, 8). The FDA neither conducts research, review experimental designs, and statistical analyses nor reaches independent conclusions about the safety of a proposed GE trait or plant. In essence, FDA has allowed companies to assert that new, “substantially equivalent” GE crops are “generally recognized as safe” (GRAS) (8).

Once so designated, officially or in practice, there is little or no justification for any ongoing, food safety-focused regulatory scrutiny, or need for federal investment in research on possible food safety risks. In short, the FDA’s process and actions suggest that the science is settled, despite the lack of modern, well-designed studies of the sort needed to detect subtle cellular, metabolic, genetic, and epigenetic impacts that do not substantially change the nutrient composition of harvested foodstuffs.

### The Environmental Protection Agency

The Environmental Protection Agency (EPA) was, and remains, responsible for the assessment and approval of GE applications accompanied by pest management-related claims. EPA science reviews and actions evolve in accord with the detailed requirements and regulations put in place over decades in administering the Federal Insecticide, Fungicide, and Rodenticide Act (FIFRA), an Act addressing chemical and botanical pesticides. The EPA’s GE-related responsibilities include

- characterizing and quantifying exposures to novel proteins or other toxins produced by GE crops;
- assessing the need for new or altered tolerance levels for GE plant proteins and/or pesticides used in conjunction with GE crops;
- determining whether a new GE application poses any new or worrisome worker or applicator risk, or environmental risks; and
- addressing the risk of resistance, and whether and how steps should be taken *via* mandatory label directions to mitigate the risk of resistance.

The EPA regulates GE microorganisms under the Toxic Substance Control Act, despite the indisputable fact that the risks stemming from release of GE microorganisms that can reproduce and spread are very different than the risks posed by toxic chemicals, which cannot reproduce.

### United States Department of Agriculture

Ironically, the United States Department of Agriculture (USDA)’s role in GE regulation is the least important relative to risk identification and prevention, but has triggered the most extensive delays, as well as the most intensely contested litigation and public controversy. The USDA regulates the agronomic and some environmental impacts of GE plants under the Plant Protection Act (PPA), a statute that limits the purview of USDA assessments to whether GE plants might act as “plant pests” (e.g., as a weed or virus) (8). Thus, if a plant is GE but does not contain genetic material from a known, plant pest, the plant is typically not considered a “regulated article” (9).

The USDA regulates GE insects under the Animal Plant Health Protection Act, which was designed to protect livestock and poultry, including farmed fish from animal diseases. Thus, for GE insects, USDA only considers whether the GE insect has an impact on communicable diseases of livestock and poultry, rather than broader environmental or ecological impacts.

## Critical Challenges Confronting the Coordinated Framework

No one expects the Coordinated Framework review process started by the Obama Administration to quickly solve any of the foundational problems with biotechnology regulation in the U.S. (4). But it will hopefully clarify the major issues and challenges, and bring new players and ideas into the ongoing policy-reform process.

I suspect that eventually the U.S. will be forced to upgrade the science supporting the assessment and management of risks arising from agricultural biotechnologies. The now-heavy dose of wishful thinking embedded in GE risk assessments will hopefully be replaced with hard science. Progress is especially needed in five areas in order to create a biotechnology regulatory framework that is as dynamic as the science and technology it seeks to help manage.

### Focus on Fetal and Child Development

To date, there has been little serious research on the impact of GE crops and technology on human reproductive performance and childhood development, despite wide recognition that untimely, very low dose pesticide and toxin exposures can trigger endocrine system and epigenetic effects of lasting consequence (10).

For this reason, it is indeed unfortunate that EPA has failed to invoke the historic, health-promoting provisions of the 1996 Food Quality Protection Act (FQPA) in its assessments of the acceptability of GE technology. The FQPA calls for an added 10-fold safety factor in regulating pesticides, and indeed any crop protection technology, when there is (a) uncertainty regarding risks to pregnant women, infants, and children or (b) inadequate data to characterize exposure levels (11).

On both of these counts, several GE technologies and their associated pesticides should have triggered the FQPA’s added safety factor. This is an area ripe for litigation.

### Gene Editing Technologies

The high-priority issues throughout the review of the Coordinated Framework will surely include how to deal with gene editing technologies, such as RNAi, and other new gene editing technologies (e.g., CRISPR-cas9, TALEN, ZNF, and meganucleases) (4, 12). Many of these gene editing techniques will presumably not entail movement of foreign deoxyribonucleic acid (DNA) into crops.

Under current policy, companies or teams using RNAi and gene editing tools can simply write to the USDA and request a letter from the Department acknowledging that the resulting GE plants are not “regulated articles.” To date, USDA has sent letters exempting over 30 GE plants from USDA reviews – including multiple glyphosate-tolerant crops and Loblolly pine trees with increased wood density.

If these new gene editing technologies are deemed exempt from U.S. regulatory reviews, as many in the GE industry have requested, these presumably safer technologies will invite intense scrutiny and likely create a new wave of litigation, market disruption, and labeling confusion.

### New Tools to Manage Adoption

The revised Framework must recognize that the scale of adoption of any GE crop technology will drive the nature and magnitude of possible adverse environmental, public health, or marketplace consequences.

Under current law and regulations, federal agencies assess the risks and benefits of a new GE technology when planted or adopted on a given field. It is assumed that the risks arising from the planting of any particular field to a GE crop will be determined solely by what happens in that field. Current risk assessments do not take into account whether a given GE technology is likely to be adopted on 1%, or 10%, or nearly 100% of the cropland planted to a specific crop.

Current policy and risk assessments also fail to consider incremental and cumulative adverse impacts that can worsen over time, such as the rise in the costs of weed management (13, 14), loss of biodiversity, and increases in the volume and number of herbicide applications that invariably follow the emergence and spread of herbicide-resistant weeds (15–17).

For technologies that depend on biological and ecological impacts and interactions to work as intended (e.g., essentially all GE crop technologies), wider and more frequent use will generally result in additional, and/or potentially more severe, unintended consequences. This general rule applies to all biologically based technologies and has stood the test of time.

Going forward, the revised Coordinated Framework must grapple with the challenge of calibrating the sophistication and sensitivity of risk assessments, and risk mitigation interventions, to the scope of adoption and the magnitude of possible and actual adverse impacts. Fortunately, there are already accepted regulatory strategies and tools in place to do so in the U.S., and several have already been invoked in approvals of GE crop technology [e.g., refuge requirements, limiting deregulation decisions (approvals) to specific geographic areas or fixed time periods, and mandatory monitoring of target insects for resistance].

The revised Framework should work toward calibrating the risk assessment process to the scope of adoption by seeking from technology developers an estimate of the expected degree of market penetration in specific regions, within say 5 years of approval. Agencies could then focus risk assessments on high-adoption regions, and if deemed necessary, limit approvals or impose targeted monitoring or risk mitigation measures. After 5 years, the agency and technology developers could then re-assess estimates of adoption, actual experiences in the field, and the need for any further efforts to better characterize or mitigate risk.

Such new tools and authority is badly needed to avoid the proliferation of collateral damage to farmers and the food industry (e.g., failing and increasingly expensive pest management systems; loss of markets) and/or the environment and public health.

### Dealing with Risks Arising from the Emergence and Spread of Resistant Organisms

The failure of the Coordinated Framework to address the risk and consequences of resistance is a serious deficiency. In fairness to the agencies implementing the Coordinated Framework in the early years, several constructive steps were taken to build resistance management into the *Bt*-transgenic corn- and cotton-approval processes. These included sizable, mandatory refuges planted to non-GE-*Bt* seeds and rigorous, annual resistance monitoring of insect populations.

After about a decade of largely successful prevention, GE technology developers pressured the EPA to relax *Bt*-crop refuge requirements, despite warnings from many independent entomologists. The consequences, and price tag, associated with this regrettable error in judgment are steadily rising and will continue rising for years to come.

To prevent resistance from eroding the benefits of GE crop technology and GE-based animal health and microbial products, the revised Framework should direct all federal agencies to take a variety of steps. The most important include

- sponsoring competitive research grant programs on the genetic mechanisms triggering resistance and/or the spread of GE technology-induced resistance;
- phasing out the use of antibiotic-related marker genes;
- requiring resistance risk assessments and management plans as a routine part of applications for approval and evaluating such plans *via* an independent review panel;
- post-approval resistance-monitoring provisions, including how ongoing resistance management testing will be paid for; and
- establishing resistance thresholds when exceeded will quickly trigger a second tier of resistance risk prevention strategies.

### Need for Independent Science

Most people expressing a view on how the Coordinated Framework needs to be updated agree on one thing – poor and inadequate science has become an endemic problem in the GE risk assessment and regulatory processes (4). The revised Coordinated Framework must broaden and deepen the science base supporting GE regulation in order to enhance confidence in the scientific judgments supporting government decisions on GE technology.

Another step is equally important in convincing those skeptical of current GE crop safety assessments, within and outside the scientific community. The majority of the new, more sophisticated risk-assessment science should be conducted by scientific teams with no ties to the companies developing and marketing GE crop technology. In addition, institutions funding and carrying out this work should take proactive steps to insulate the individuals conducting the work from non-scientific criticisms, personal and professional attacks, and initiated or supported by GE companies and their surrogates. Regrettably, such unprecedented measures are now needed to slow the erosion of scientific integrity in this economically important, fast-moving area of technology.

Several practical, low-cost steps can be taken immediately. Federal agencies should require, as part of the application process, a guarantee from technology developers that requests for isolines and/or genetic markers and probes, or other technical information necessary to conduct risks assessments will be provided to federal agency scientists and independent researchers, and without imposition of restrictions on what non-commercial research can be conducted with them, or when and how results may be reported.

The FDA should publicly disclose the data provided by GE technology developers and allow for public comments on these data as well as on the adequacy of risk assessments. Both steps should be completed before the GE organisms are allowed on the market. Approvals can then incorporate any needed actions and requirements, such as post-approval surveillance and additional testing for applications in areas outside those studied prior to commercial launch.

Before any field trial of a new GE trait, USDA should require and disclose the exact sequence information of the inserted genetic material so that USDA, the grain trade, and food companies can detect possible contamination. Presently, USDA does not require detailed sequence information and therefore has no way to detect contamination. In addition, the locations of GE field trials should be disclosed, so that neighboring farmers and the food industry can guard against genetic contamination.

Independent scientists should be awarded funding and granted both the needed time and data/tools necessary to conduct state-of-the-art, GE technology risk assessments. They must be free to raise questions and reach independent conclusions without fear of personal or professional retaliation. Efforts to elucidate the metabolic-breakdown pathways of novel proteins in the edible portions of GE plants should receive special focus and dedicated funding, now that some widely consumed, GE fresh fruits (e.g., Artic apple) and vegetables (Innate potatoes, and *Bt* and Roundup Ready sweetcorn) have been approved and are in the food supply in several countries.

## Toward a Brighter Future of Biotech Regulation in the U.S.

Currently in the U.S., the trigger for GE regulatory oversight is based on the attributes of GE organisms not the process used to create them (e.g., GE *via* a gene gun). This is conceptually flawed and leads to all sorts of problems: the USDA exempts GE plants produced without plant pest DNA from its admittedly limited purview; the FDA allows GE plants to be treated as GRAS if deemed substantially equivalent, with little or no focus on novel risks that are outside the parameters considered in judging substantial equivalence. It also means that some of the risks associated with GE organism (genetic contamination, resistant pests) are simply neither assessed nor addressed.

Five steps discussed below address specific, concrete steps the U.S. federal government could take to improve the GE risk assessment and risk mitigation processes. Steps 3.1 and 3.5 could be adopted relatively quickly, while more time to craft and implement solutions will be necessary in the case of the other three.

### Adopt the Internationally Accepted Definition of Biotechnology

The U.S. should adopt the definition of “modern biotechnology” set forth by the Codex Alimentarius in the *Principles for the Risk Analysis of Foods Derived from Modern Biotechnology* (18):

‘Modern biotechnology’ entails the application of:

(i) *In vitro* nucleic acid techniques, including recombinant deoxyribonucleic acid (DNA) and direct injection of nucleic acid into cells or organelles, or (ii) Fusion of cells beyond the taxonomic family, that overcome natural physiological, reproductive, or recombinant barriers and that are not techniques used in traditional breeding and selection.

### Assess All Aspects of GE Applications

The new Coordinated Framework should direct federal agencies to take into account both the novel proteins and other compounds produced by a GE plant, as well as any other related chemicals that must be, or typically will be used in conjunction with the GE crop technology. These will, of course, include all herbicides associated with a herbicide-tolerant crop variety, as well as seed treatments marketed as important in order for farmers to bring a GE crop to harvest.

### Restore Scientific Integrity in Judging Substantial Equivalence

Despite its flaws, “substantial equivalence” is likely to remain part of the GE regulatory process. Accordingly, the erosion of scientific integrity in the assessment of GE crop equivalence must be reversed by

- Assuring that the protein, trait, or plant under investigation in risk-assessment studies is identical to those from the GE plant under evaluation;
- No longer considering the range of “natural variation” in nutrient and phytochemical levels in a GE crop versus its isoline, when both are grown in properly designed side-by-side trails;
- Requiring that the diet fed to control animals consists of the isoline of the GE crop being tested, and the GE crop and its isoline should be grown in the same environment; and
- The diet of the control animals should be tested for the presence of contamination from other GE crops and pesticides typically used in conjunction with GE crops.

### Acknowledge That Stacked Varieties May Pose Unique Risks

The new Framework should require agencies to develop new test requirements for stacked varieties, acknowledging that multiple traits and regulatory sequences can lead to unexpected interactions and possibly adverse outcomes, just as treatment with multiple medications can lead to drug interactions and contraindications.

### Require Labeling of GE Products

All products from GE crops, animals, and microorganisms should be accurately labeled, both to ensure consumer choice and to enhance the odds that public health officials, doctors, and scientists will quickly recognize unexpected problems, if and when they arise. In addition, companies that develop GE organisms should be required to disclose any GE trait, marker genes, or other genetic constructs in commercial, GE seed products, including traits and genes from obsolete and no longer-marketed traits.

Today, FDA requires labeling on food products when there has been a change in a “material fact,” such as a food product’s nutritional value, organoleptic properties, or functional characteristics. But several times in the past, and for good reason, the FDA has required labeling under the “material fact” construct in the absence of a change in nutritional value, organoleptic properties, or functional characteristics.

For example, in the final food irradiation rule, the FDA acknowledged that the large number of respondents who asked for labeling of irradiated retail products was evidence that irradiation was, indeed, a “material fact” (19). In its decision, the FDA wrote “Whether information is material under section 201(n) of the act depends not on the abstract worth of the information but on whether consumers view such information as important and whether the omission of label information may mislead a consumer. *The large number of consumer comments requesting retail labeling attest to the significance placed on such labeling by consumers*” (Emphasis added) (19).

Clearly, state and national polling, and the near 50–50% split in the voting on several state GE food ballot initiatives, is evidence of the significant consumer interest in whether a food product contains GE ingredients.

## Author Contributions

The author confirms being the sole contributor of this work and approved it for publication.

## Conflict of Interest Statement

CB is a member of the U.S. Department of Agriculture’s AC 21 Agricultural Biotechnology Advisory Committee. He serves as an expert witness in several lawsuits involving the labeling of foods derived from genetically engineered crops.
